# Association between WASH-Related Behaviors and Knowledge with Childhood Diarrhea in Tanzania

**DOI:** 10.3390/ijerph18094681

**Published:** 2021-04-28

**Authors:** Natalie Bennion, Generose Mulokozi, Emily Allen, Margaret Fullmer, Gwen Kleinhenz, Kirk Dearden, Mary Linehan, Scott Torres, Joshua West, Benjamin Crookston, Cougar Hall

**Affiliations:** 1 Department of Public Health, Brigham Young University, LSB, Provo, UT 84602, USA; emily.d.allen8@gmail.com (E.A.); fullmerster@gmail.com (M.F.); gwenellenkleinhenz@hotmail.com (G.K.); west.josh@gmail.com (J.W.); benjamin.crookston@gmail.com (B.C.); cougar_hall@byu.edu (C.H.); 2 IMA World Health, Nyalali Curve, PO Box 9260, Plot 1657, Dar es Salaam, Tanzania; generose.mulokozi@gmail.com; 3 IMA World Health, 1730 M St NW #1100, Washington, DC 20036, USA; kdearden@imaworldhealth.org (K.D.); marylinehan609@gmail.com (M.L.); 4 RTI International, 701 13th St NW #750, Washington, DC 20005, USA; torscott@gmail.com

**Keywords:** WASH, diarrhea, knowledge, behavior, communication campaign

## Abstract

Background: Diarrhea remains a major cause of morbidity and mortality among children in Tanzania. The purpose of this study was to explore associations between diarrheal disease and water, sanitation, and hygiene (WASH) related behaviors and determine care-seeking predictors for diarrheal disease. Methods: Data from 9996 female primary caregivers were collected as part of a larger integrated nutrition program. Logistic regression was used to measure associations between predictor and dependent variables and diarrheal and care-seeking outcomes. Results: Knowledge of the importance of handwashing after assisting a child who has defecated (OR 0.79, CI 0.72–0.87), before preparing food (OR 0.88, CI 0.80–0.97), and before feeding a child (OR 0.89, CI 0.81–0.99) were each associated with not having a child with diarrhea in the past two weeks. Fathers or male caregivers (OR 0.65, CI 0.48–0.89) were less likely to seek medical care for a child with diarrhea. No associations were found between WASH-related knowledge or behavior and seeking medical care for a child with diarrhea. Conclusions: Findings indicate that knowledge of handwashing importance was significant in washing hands after assisting a child who has defecated, before preparing food, and prior to feeding a child. These findings demonstrate the value of parental involvement to lower morbidity and mortality among children.

## 1. Introduction

Despite significant declines worldwide, diarrhea remains a major cause of morbidity and mortality among children in developing countries. In 2010, 64% (5.5 million) of the 7.6 million deaths in children under the age of five years old were attributable to infectious diseases and 10.5% were caused by diarrhea [1]. While the number of children suffering from diarrhea is largest in South Asia, the highest prevalence of childhood deaths from diarrhea is in Sub-Saharan Africa where 50% of all diarrhea-related deaths occur [2]. The high disease prevalence of diarrhea in East Africa is correlated with poor hygiene, education level of the household head, and obtaining water from surface sources or wells and per-capita water use for cleaning [3]. In Tanzania, diarrhea is the leading cause of childhood death. One-third of deaths in Tanzanian children under five years of age are related to poor hygiene, with the majority of these deaths due to diarrhea [4]. Safe stool removal, age of the mother, residing in an informal settlement, as well as the mother’s handwashing during meal preparation and following the changing of children’s diapers were significantly associated with self-reported diarrhea among children less than five years of age during the previous 14 days [5,6].

A study in three East African countries identified an increased risk of diarrhea associated with unimproved sanitation and having to travel more than 30-min to a water source. Long distances to water sources prompt families to store water for long periods of time, leading to possible water contamination and the negative effects associated with it [7,8]. Water, sanitation, and hygiene (WASH) behaviors are important factors in reducing rates of diarrheal diseases and child mortality [9,10].

Lack of access to basic water supply is a major problem in both rural and urban Tanzania [11]. Less than half of the rural population in Tanzania has access to safe drinking water [12]. Access to clean and safe water in rural areas declined from 46% to 40% between 2001 and 2007 [13]. The limited availability of water in Tanzania can inhibit WASH-related behaviors, especially handwashing practices [10].

Along with WASH-related behaviors and diarrhea, it is important to consider care-seeking predictors for diarrheal disease. A study on social determinants of care-seeking in Peru found that rural residence was significantly associated with seeking care for diarrhea. Water source, maternal education and wealth were also significant predictors of seeking care for diarrhea [14]. Rural communities in South Africa may indicate spiritual and cultural beliefs as being a predictor of receiving care [15]. In Tanzania, predictors including mother’s occupation, wealth status, distance to health facilities, child’s age and place of residence were all significant factors of whether a caregiver sought medical care for a child’s diarrhea [16].

WASH-related programming in Tanzania has been correlated with a reduction in WASH-related diseases [17]. Diarrhea-specific mortality rates (DSMR) among children under five years decreased by 89% from 1980 to 2015 in Tanzania, largely due to key childhood interventions targeting WASH behaviors. An increased understanding of both the predictors of WASH-related behaviors and care-seeking behaviors for children with diarrhea are key to scaling up interventions with the ability to further reduce DSMR in Tanzania [17]. The purpose of this study was two-fold: first, to explore associations between WASH-related behaviors and knowledge and diarrheal disease and; secondly, to identify significant care-seeking predictors for diarrheal disease.

## 2. Materials and Methods

### 2.1. Design

An evidence-based communication campaign was implemented between 2015 to 2020 in five regions of the Lake Zone in Tanzania. The purpose of the campaign was to prevent childhood stunting through the promotion of optimal health behaviors and knowledge regarding WASH and community nutritional practices. The communication campaign included support groups, mobile outreach clinics, home visits, WASH intervention training, and a media campaign that was broadcasted via radio and television between June 2017 and March 2020. This study does not report directly on the effects of the campaign or any of its related components, but instead opportunistically uses the cross-sectional data gathered for the campaign and examines associations between WASH-related knowledge and behaviors, as well as diarrheal outcomes.

### 2.2. Study Design

A survey was developed by the Addressing Stunting in Tanzania Early (ASTUTE) program to examine trends in key maternal and child health related behaviors. Eligibility for participation included households with children aged 0–23 months. Questions were directed to female caregivers. The survey contained 169 questions and took an average of 50–60 min to complete. The survey was written in English and translated to Kiswahili by Ipsos, a data collection firm. Pilot testing occurred prior to survey administration.

### 2.3. Data Collection and Sampling

Data were collected by a field team consisting of 10 supervisors and 50 enumerators. Data collection occurred digitally via smartphones and personal digital assistants (PDAs). The National Institute for Medical Research in Tanzania and relevant local government authorities authorized the research (NIMR/HQ/R.8a/Vol.IX/2344). Institutional Review Board (IRB) approval was obtained through an internal IRB at Developmental Medial International (DMI), a research and communications not-for-profit organization that implemented mass media activities. Quality checks were verified by 11 quality controllers and new interviews were conducted when the quality of a completed interview could not be validated.

Prior to the intervention, villages were randomly selected (*n* = 243) from five regions (Geita, Kagera, Kigoma, Mwanza, and Shinyanga) of the Lake Zone. A stratified, multi-staged random sample design was used to select survey participants. Participants within each village were randomly sampled anew during each survey period. All participation was voluntary and required informed consent. Data was collected from 9996 households from 2017 through 2020.

### 2.4. Statistical Methods and Analysis

This study examined household and demographic information. All major variables used were dichotomous. Dependent variables included whether the child experienced diarrhea within the past 2 weeks and if the child received medical treatment for the diarrhea. Behavioral variables used were optimal stool disposal, access to a water source, how frequently the household washes hands with soup, if a handwashing station exists in the home, if there is soap or ash at the handwashing station, if there is water at the handwashing station, and if the household owns their own soap. Knowledge variables used were based upon knowledge of when it is important to wash one’s hands including after latrine use, after assisting child who has defecated, before preparing food, before eating food, and before feeding a child as well as knowledge on whether or not handwashing with water alone makes one’s hands clean. Sociodemographic-related predictor variables used included number of children, sex of the child, relationship to the child, mother’s education, household wealth, child’s age, and mother’s age.

Optimal stool disposal was determined by whether the child used latrine, put stool into a latrine, or threw stool into the garbage. Poor stool disposal included putting it in a ditch, buried, left in the open, or unknown. The wealth index was constructed using a similar approach to wealth measurement used by Briceño [18]. In brief, the wealth index included access to safe drinking water sources, access to safe sanitation, as well as ownership of a radio, television, bicycle, motorcycle, automobile, mobile phone, boat, and/or animal-drawn cart. Relationship of the caregiver was re-coded into three variables denoting mother, father, and other (i.e., grandmother, grandfather, sibling, aunt/uncle). This decision-maker variable was composed of one’s self, partner, or both as equal decision makers.

Logistic regression was used to examine the association between WASH practices and diarrhea and also the association between predictor variables and whether an individual sought out medical care for diarrheal disease. Adjusted models controlled for maternal age, maternal education level, and household wealth. Dependent variables included whether or not the child had diarrhea within the past 2 weeks and if the child received medical care for the diarrhea. Risk factors included WASH behaviors exhibited by the primary caregiver including handwashing techniques and method of stool disposal. Predictor variables (key indicators) included maternal age, child age, number of children, child gender, maternal education, maternal literacy, which parent stays home when the child is sick, and who makes decisions on healthcare. Odds ratios, *p*-values (a = 0.05) and 95% confidence intervals were used to assess the strength of the associations. Significant findings were determined by <0.05 *p*-value. Data were examined using SAS 9.4 software (SAS Institute, Cary, NC, USA). Hosmer and Lemeshow goodness of fit tests were computed for all models and only those models that met assumptions for fit were retained.

## 3. Results

A total of 9996 households participated in the study (see Table 1). Median age for respondents was 28.2 years old and 56.5% of mothers had completed primary school. The majority of participants were married (81.8%) and lived in an urban setting (86.0%). Children represented by study households were evenly split between male (49.7%) and female (50.3%).

Approximately one-quarter (23.2%) of caregivers reported a child having experienced diarrhea within the past two weeks and 74.8% of those who had diarrhea sought medical care for childhood diarrhea (see Table 2). The majority of caregivers (86.3%) reported optimal child stool disposal and access to an optimal water source (65.1%). Few caregivers had access to handwashing stations with soap or ash (49.0%) and water (58.4%) being available at the stations. The majority of caregivers (82.5%) reported that handwashing is important after latrine use and before eating (73.3%), with fewer reporting the importance of handwashing after assisting a child who has defecated (43.7%), before preparing food (50.0%), and before feeding a child (39.5%). Most noted that handwashing with water-only does not make hands clean (89.3%).

Knowledge of the importance of handwashing after assisting a child who has defecated (OR 0.79, CI 0.72–0.87), before preparing food (OR 0.88, CI 0.80–0.97), and before feeding a child (OR 0.89, CI 0.81–0.99) were each associated with not having a child with diarrhea in the past two weeks (see Table 3).

Parental WASH behaviors and knowledge had no association with care-seeking behaviors for when a child had diarrhea (see Table 4). Household wealth (OR 2.20, CI 1.12–4.29) and maternal education (OR 1.62, CI 1.16–2.27) were associated with care-seeking for a child with diarrhea. Fathers or male caregivers (OR 0.65, CI 0.48–0.89) were less likely to seek care for a child with diarrhea (see Table 5). 

## 4. Discussion

This study sought to understand potential associations between WASH-related behaviors and knowledge with diarrheal disease. Additionally, this study examined potential care-seeking predictors for diarrheal disease among caregivers in the Lake Zone region of Tanzania. The frequency of reported diarrhea in the past 2 weeks in this study is similar to other studies [4,5,6]. Furthermore, the percentage of caregivers seeking care for children with diarrhea found in this study is consistent with other Tanzanian samples [16].

Of particular note, the current study did not find an association between reported or observed WASH behaviors and childhood diarrhea. Participants self-reported about disposal of a child’s stool and access to optimal water sources. Other WASH behavior indicators (i.e., there is a water station; soap at a water station; owns soap) were observed at the time of survey administration. It may be that a study methodology inclusive of additional WASH behavior observation may have yielded different results. The environment and availability of resources like soap and water often plays a role in health outcomes. In Africa, more than two-thirds of the population have to leave their home in order to fetch water for drinking and domestic use. In a survey performed in 26 countries in Africa, a 15-min decrease in one-way walk time from a water source was associated with a 41% relative reduction in diarrhea prevalence in children under five years old [19]. Another study found that many households prefer to use low-quality water sources that are close by as opposed to high-quality water sources further away. Access and convenience to water sources both play a role in decreasing diarrhea prevalence and overall mortality in children. However, the current study found no significant association with diarrhea and environmental factors including access to a water source, frequency of handwashing, existing handwashing stations, and soap, ash, or water at the station. Further research is needed to better understand the environment related to distance to water sources, and frequency of use related to diarrhea outcomes in Tanzania.

This study did find several significant associations between WASH knowledge and childhood diarrhea. The five critical times for handwashing according to the World Health Organization (WHO) include: (1) after defecation (latrine use); (2) after assisting a child who has defecated; (3) following the handling of a child’s feces; (4) before preparing food, before eating food; and (5) before feeding others (child) [20]. This study found that having a knowledge of the importance of three of these handwashing times was associated with reduced childhood diarrhea. These significant times included after assisting a child who has defecated, before preparing food, and before feeding a child. Results of the current study align with portions of an earlier study conducted in Tanzania which identified washing hands before a meal as a correlate to reduced diarrheal disease in children [8]. Similarly, a study in Ethiopia identified the time periods of before preparing food and after defecation (latrine use) as the most critical for handwashing to reduce diarrhea in children [19]. Other studies regarding handwashing in Tanzania focused on general aspects of handwashing and on the use of soap and hand sanitizer rather than on knowledge of critical handwashing times [17]. These findings help indicate the critical nature of handwashing before preparing food, before childhood feeding, and after defecation in reducing diarrhea in children, but more studies linking knowledge of critical handwashing times, rather than general WASH practices, need to be conducted in Tanzania. Such efforts could validate the results of this study and further explore the potential impact that knowledge of all five critical handwashing times may have on childhood diarrhea.

This study explored predictors of seeking care for childhood diarrhea. Understanding associations with care-seeking behaviors and diarrheal disease is important for improving childhood health outcomes. Wealth status has consistently been shown to have a positive impact on care-seeking behavior. A study examining determinants of care-seeking behavior in Nairobi reported that the wealthiest families seek outside medical care more than 2.5 times as often as the poorest families [20]. Further, two-parent households, especially those including a mother who generated her own income, were more likely to seek out-of-the-home medical care for their children. Consistent with the literature, household wealth and caregiver education were both predictive of seeking care for a child with diarrhea in the current study. Other knowledge and behavior characteristics including method of stool disposal, having access to water source, knowing when it is important to wash one’s hands, and both the knowledge and behavior of washing hands with soap were not directly associated with seeking care for a child with diarrhea [20]. These findings further support the current literature that the most critical predictors of care-seeking behaviors are household wealth, gender, and education [21,22].

Mothers were significantly more likely to seek care for a child with diarrhea than fathers in the current study. This finding is consistent with previous research. Bedford and Sharkey identified gender dynamics as a key factor in care-seeking decisions in Nigeria [23]. The authors concluded that fathers making care-seeking decisions was a barrier to accessing effective, efficient and timely healthcare [23]. Several studies examining care seeking behaviors in various African nations have noted that fathers are typically in charge of deciding what types of care to choose—decisions most often impacted by financial costs [22,24]. Both Bedford and Sharkey [23] and Bakshi [24] noted distinct cultural and geographic differences between groups in the gendered dynamics of care-seeking decisions, with some regions being more culturally traditional and patriarchal and others engaging in an increasingly collaborative decision-making process. Funk [25] indicated differences in perceived responsibility for childcare among fathers, specifically noting differences between traditional fathers who consider childcare to be the mother’s responsibility, transitional fathers who view childcare as a shared responsibility depending upon the context, and modern fathers who consider childcare to be a shared responsibility regardless of circumstance. It appears that socioeconomic factors together with cultural and regional norms impact a father’s decision of whether or not to seek care for his child. While the care-seeking behaviors of male caregivers were significantly different than female caregivers in this sample, the current study is unable to determine why fathers were less likely to seek care for children with diarrhea.

It has been widely recognized that male involvement in maternal health is important for improving maternal and newborn health [26]. Early paternal involvement has been found to be beneficial for the neurodevelopment of infants, as well as maternal and newborn care [26,27]. Further, father absence is associated with lower food security, poverty and poor health outcomes [27]. Healthcare professionals strive to encourage fathers to be involved as much, and as early, as possible, but have had limited success. In Tanzania, it has been cited that problems associated with male involvement are partly due to the position of men in society and to health systems challenges in accommodating men [28,29,30]. For long-term childhood health, implementation of effective strategies that mitigate cultural and structural challenges, as well as encourage male involvement in the home, are essential. Panter-Brick, Burges, and Eggerman conducted a systematic review of the global literature on parenting interventions [30]. They determined three key priorities that must be a part of any parenting intervention to encourage father involvement: (1) engage couples together; (2) separate the process and impact by fathers, mothers, and coparents; and (3) pay more attention to the issues of reach, sustainability, cost, equity, and scale-up [30]. A community-based intervention to educate with the Home-Based Life Saving Skills (HBLSS) program has been shown to successfully address these three key priorities. This intervention consists of community education through trained community health workers [25,26,27,28]. Both August et al. and Sibley et al. found HBLSS to be feasible, scalable and effective in improving male involvement in maternal healthcare in Tanzania [27,31]. Results from August et al. showed a significant overall improvement in male involvement (39.2% vs. 80.9%) [27]. Sibley et al. found HBLSS to increase access to basic life saving measures within the home, the community, as well as decreasing delays in reaching referral facilities where life-threatening problems can be managed appropriately [31].

Results from the current study should be considered in light of several key limitations. First, this study was cross-sectional and therefore unable to demonstrate a causal relationship between study variables. Second, this study explored associations between self-reported WASH behaviors and knowledge on childhood diarrhea and did not include extensive observation of actual participant WASH behaviors. Third, the data analyzed do not represent all of Tanzania, but rather represent five regions of the Lake Zone. Despite these limitations, this research is still highly relevant as prevention of childhood diarrhea in Tanzania is a public health priority. The current study’s large sample size and rigorous methodology strengthen the impact of its key findings and add to a growing body of literature helpful in designing programs aimed at improving childhood health outcomes through the prevention of diarrhea and stunting.

## 5. Conclusions

In conclusion, caregiver WASH knowledge is positively associated with decreased childhood diarrhea while care-seeking behavior for childhood diarrhea is positively associated with household wealth and maternal education and negatively associated with male caregivers. These key findings demonstrate the need to strengthen ongoing promotion of WASH-related knowledge in Tanzania, particularly health promotion and education related to critical times for handwashing. Efforts to engage and include husbands, fathers, or other male caregivers in maternal and child health are essential. Those aiming to prevent, or address childhood diarrhea and stunting, can use these findings to inform their program planning.

## Figures and Tables

**Table 1:** Participant demographics.

Demographics	N (%)/Mean (SD)
Maternal age	28.2 (7.08)
Child age	N (%)
0–6 months	3945 (39.76)
7–12 months	2638 (26.59)
13–18 months	2093 (21.09)
19–23 months	1246 (12.56)
Child gender	
Female	5025 (50.27)
Male	4971 (49.73)
Education	
Less than primary	3027 (30.28)
Completed primary	5648 (56.51)
Secondary or more	1320 (13.21)
Marital Status	
Single	504 (5.04)
Married (monogamous)	7399 (74.02)
Married (polygamous)	781 (7.81)
Informal union	500 (5.00)
Widowed	161 (1.61)
Divorced	45 (0.45)
Separated	601 (6.01)
Other	5 (0.05)
Setting	
Urban	8597 (86.00)
Rural	1399 (14.00)

**Table 2:** Key indicator frequencies (N = 9996).

Outcomes	N (%)
Had diarrhea within the past 2 weeks	2322 (23.23)
Sought care for diarrhea	1763 (74.76)
Behaviors	
Optimal stool disposal ^a^	8629 (86.33)
Access to optimal water source	6505 (65.08)
There is soap or ash at the station	527 (48.98)
There is water at the station	634 (58.92)
Has/owns own soap	2136 (45.22)
Knowledge	
Handwashing techniques—When is it important to wash your hands?	
After latrine use	8244 (82.47)
After assisting child who has defecated	4365 (43.67)
Before preparing food	5001 (50.03)
Before eating food	7325 (73.28)
Before feeding a child	3945 (39.47)
Handwashing with water alone	1165 (11.65)
Sociodemographic factors	
Maternal literacy	7587 (76.16)
Adult stays home while the child is ill	1502 (76.28)
Who makes decisions on health care?	
Both parents	1762 (29.10)
Self	2327 (38.42)
Partner/spouse	1967 (32.48)

^a^ Optimal stool disposal includes child used latrine, put stool into a latrine, or disposed of stool in the garbage. Poor stool disposal includes being put into a ditch, buried, left in the open, other, or unknown.

**Table 3:** Regression analysis for parental WASH behavior and knowledge and the association with child diarrhea during past 2 weeks (N = 9996).

Outcomes	N (%)
Had diarrhea within the past 2 weeks	2322 (23.23)
Sought care for diarrhea	1763 (74.76)
Behaviors	
Optimal stool disposal ^a^	8629 (86.33)
Access to optimal water source	6505 (65.08)
There is soap or ash at the station	527 (48.98)
There is water at the station	634 (58.92)
Has/owns own soap	2136 (45.22)
Knowledge	
Handwashing techniques—When is it important to wash your hands?	
After latrine use	8244 (82.47)
After assisting child who has defecated	4365 (43.67)
Before preparing food	5001 (50.03)
Before eating food	7325 (73.28)
Before feeding a child	3945 (39.47)
Handwashing with water alone	1165 (11.65)
Sociodemographic factors	
Maternal literacy	7587 (76.16)
Adult stays home while the child is ill	1502 (76.28)
Who makes decisions on health care?	
Both parents	1762 (29.10)
Self	2327 (38.42)
Partner/spouse	1967 (32.48)

Logistic regression adjusted for maternal age, maternal education level, and household wealth. All models satisfied assumptions for goodness of fit. ^a^ Optimal stool disposal includes child used latrine, put stool into a latrine, or disposed of stool in the garbage. Poor stool disposal includes being put into a ditch, buried, left in the open, other, or unknown.

**Table 4:** Regression analysis for parental WASH behavior and knowledge with care-seeking behaviors for diarrhea (N = 9993).

	Odds Ratio (CI)	*p*-Value
Behaviors		
Optimal stool disposal ^a^	1.06 (0.92–1.22)	0.45
Access to a water source	1.10 (0.91–1.32)	0.33
Frequently washes hands with soap and water	0.92 (0.81–1.10)	0.24
There is soap or ash at handwashing station	1.21 (0.87–1.69)	0.25
There is water at the station	1.1 (0.79–1.52)	0.61
Household has/owns own soap	1.14 (1.0–1.31)	0.07
Knowledge/attitudes		
Times it is important to wash your hands		
After latrine use	1.03 (0.91–1.17)	0.65
After assisting child who has defecated	0.79 (0.72–0.87)	<0.0001
Before preparing food	0.88 (0.80–0.97)	0.01
Before eating food	1.02 (0.92–1.1)	0.69
Before feeding a child	0.89 (0.81–0.99)	0.02
Handwashing with water alone makes your hands clean	1.01 (0.86–1.17)	0.98

Logistic regression adjusted for maternal age, maternal education level, and household wealth. All models satisfied assumptions for goodness of fit. ^a^ Optimal stool disposal includes child used latrine, put stool into a latrine, or disposed of stool in the garbage. Poor stool disposal includes being put into a ditch, buried, left in the open, other, or unknown.

**Table 5:** Regression analysis for variables associated with care-seeking behaviors for diarrhea (N = 1763).

Predicting Care-Seeking Behaviors	Odds Ratio (CI)	*p*-Value
Maternal age (years)	1.00 (0.98–1.01)	0.77
Child age (months)	1.01 (1.0–1.03)	0.10
Household wealth ^a^	2.20 (1.12–4.29)	0.02
Number of children	0.97 (0.86–1.10)	0.63
Maternal education		
Less than primary school	-	-
Completed primary school	1.17 (0.95–1.45)	0.45
Some secondary education or more	1.62 (1.16–2.27)	0.01
Relationship between primary caregiver and child		
Mother	-	-
Father	0.65 (0.48–0.89)	0.02
Other	1.08 (0.67–1.75)	0.24
Maternal literacy	0.93 (0.69–1.25)	0.62
Female child	0.89 (0.73–1.08)	0.22
Adult stays home with sick child	0.95 (0.57–1.58)	0.85
Who makes decisions on health care?		
Both	-	-
Self	0.82 (0.61–1.10)	0.27
Partner/spouse	0.88 (0.64–1.22)	0.85
Optimal stool disposal behavior ^b^	0.84 (0.62–1.13)	0.24
Household has/owns own soap	1.01 (0.77–1.33)	0.93

Logistic regression models adjusted for maternal age, maternal education level, and household wealth. All models satisfied assumptions for goodness of fit. ^a^ Wealth index based on total assets. Total assets include consumer durables the individual owns including whether or not they have safe water supply and safe sanitation service. Values range from 0 to 1 where 0 is not wealthy (least assets) and 1 is most wealthy (most assets). ^b^ Optimal stool disposal includes child used latrine, put stool into a latrine, or disposed of stool in the garbage. Poor stool disposal includes being put into a ditch, buried, left in the open, other, or unknown.

## Data Availability

Restrictions apply to the availability of this data. Data was obtained from IMA World Health and contractual obligations state that data cannot be shared by the authors.
